# Concordância entre Framingham, Pooled Cohort Equations e Globorisk-LAC da estimação do risco cardiovascular no Brasil, 2013

**DOI:** 10.36660/abc.20240405

**Published:** 2025-07-01

**Authors:** Leonardo Ferreira Fontenelle, Thiago Dias Sarti, Gabriela Callo Quinte, Ana Paula Santana Coelho Almeida, José Geraldo Mill

**Affiliations:** 1 Universidade Federal do Espírito Santo Departamento de Medicina Social Vitoria ES Brasil Universidade Federal do Espírito Santo (UFES) Departamento de Medicina Social, Vitoria, ES – Brasil; 2 Universidade Federal do Espírito Santo Programa de Pós-Graduação em Saúde Coletiva Vitoria ES Brasil Universidade Federal do Espírito Santo (UFES) Programa de Pós-Graduação em Saúde Coletiva, Vitoria, ES – Brasil; 3 Universidade Federal do Espírito Santo Programa de Pós-Graduação em Ciências Fisiológicas Vitoria ES Brasil Universidade Federal do Espírito Santo (UFES) Programa de Pós-Graduação em Ciências Fisiológicas, Vitoria, ES – Brasil

**Keywords:** Fatores de Risco de Doenças Cardíacas, Brasil, Medição de Risco

## Abstract

**Fundamento:**

Os escores de Framingham e Pooled Cohort Equations (PCE) nunca foram recalibrados para a população brasileira. O *Globorisk-LAC* foi derivado recentemente com uma metodologia análoga ao PCE, tendo sido recalibrado para os países da América Latina.

**Objetivos:**

Descrever a concordância entre os escores de Framingham, PCE e *Globorisk-LAC* na estimação do risco cardiovascular em dez anos na população brasileira.

**Métodos:**

Neste estudo de corte transversal, o risco foi estimado pelos três escores para pessoas com idade entre 40–74 anos sem doença cardiovascular, usando dados da Pesquisa Nacional de Saúde (PNS) 2013. A concordância foi estimada como o percentual de casos em que o risco estimado por um escore estava entre 0,8 e 1,25 vezes o risco estimado pelo outro, e como o coeficiente de Gwet (AC_1_) para o risco estratificado em baixo, intermediário e alto.

**Resultados:**

O estudo incluiu 4.416 pessoas, entre as 8.952 do componente laboratorial da PNS. O risco mediano (intervalo interquartis) foi de 9,2% (5,1 a 17,8) pelo Framingham, 3,6% (1,7 a 8,2) pelo PCE, e 4,7% (2,8 a 8,1) pelo *Globorisk-LAC*. O risco estimado pelo Framingham concordou com o *Globorisk-LAC* em 6,4% dos casos e com o PCE em 1,8%; o PCE concordou com o *Globorisk-LAC* em 34,7%. Considerando a estratificação do risco, o AC_1_ foi respectivamente 0,454, 0,489 e 0,874.

**Conclusões:**

Os diferentes escores de risco cardiovascular concordam muito pouco entre si. Os motivos para essa discordância fazem do *Globorisk-LAC* uma ótima opção para suceder o Framingham nas diretrizes brasileiras de dislipidemia.

## Introdução

A estratificação do risco cardiovascular é amplamente recomendada por diretrizes clínicas^1-5^ como uma forma de dosar a abordagem medicamentosa da dislipidemia e outros fatores de risco. Tipicamente, ela começa alocando pessoas com doença cardiovascular a níveis de risco mais elevados. No caso das pessoas sem doença estabelecida, a estratificação envolve aplicar pontos de corte clinicamente relevantes a uma estimativa de risco cardiovascular (probabilidade de um evento numa escala de tempo de dez anos), que por sua vez é obtida através de algum escore prognóstico.

Assim como os demais modelos prognósticos, os escores de risco cardiovascular devem ter sua calibração e discriminação validadas na população onde serão usados. No estudo*Prospective Urban Rural Epidemiology* (PURE), por exemplo, os eventos cardiovasculares foram mais frequentes nos países de baixa e média renda, mesmo eles tendo escores menores do que os de alta renda.^6^ Devido a essa situação, os escores são frequentemente recalibrados para as populações onde serão usados.^6,7^ De fato, a facilidade de recalibra-los para os países-alvo é um motivo importante para a recomendação de determinados escores em algumas diretrizes.^1,3^

As mais recentes diretrizes brasileiras de dislipidemia^4,8^ recomendam o escore de risco cardiovascular global de Framingham.^9^ Já as diretrizes americanas recomendam o *Pooled Cohort Equations* (PCE),^5^ que uniu Framingham a outras coortes do mesmo país, recalibrando o resultado final para a população contemporânea dos Estados Unidos.^10^ Infelizmente, nem o escore de Framingham, nem o PCE foi recalibrado para a população brasileira.^11^ Os únicos escores de risco cardiovascular calibrados para o Brasil são os da Organização Mundial da Saúde (OMS),^12^ o *Globorisk*,^13,14^ e o *Globorisk-LAC*.^15^

Em uma pesquisa brasileira sem representatividade populacional,^16^ todos os escores examinados superestimaram o risco cardiovascular (inclusive OMS e *Globorisk-LAC*), apesar de manterem a capacidade de discriminação semelhante àquela encontrada em suas validações iniciais. Dentre os dois escores, o da OMS tem a vantagem de ter superestimado menos o risco cardiovascular naquele estudo. Enquanto isso, o *Globorisk-LAC* tem o diferencial de ter sido derivado de coortes latino-americanas,^17^ e sua versão calibrada para os países teve um desempenho semelhante, e possivelmente um pouco melhor do que o escore da OMS quando aplicados às mesmas coortes de base populacional.^15^

Como nenhum estudo até o momento avaliou a concordância entre o *Globorisk-LAC* e outros escores de risco cardiovascular, fica a dúvida de quanta diferença faria trocar o Framingham pelo *Globorisk-LAC* nas diretrizes brasileiras de dislipidemia.^4,8^ O objetivo deste estudo foi, então, avaliar a concordância entre os escores de Framingham, PCE e *Globorisk-LAC* na estimação do risco cardiovascular para a população do Brasil.

## Métodos

### Delineamento

Este estudo descritivo de delineamento transversal utilizou dados da Pesquisa Nacional de Saúde (PNS) 2013.^18^ Conduzida entre agosto e dezembro do referido ano, a pesquisa foi um inquérito domiciliar realizado em três etapas. Os conglomerados (setores censitários) foram selecionados aleatoriamente, com probabilidade proporcional ao número de domicílios particulares permanentes. Dentro destes, os domicílios (dentro dos conglomerados) e um morador com 18 anos de idade ou mais (dentro de cada domicílio) foram selecionados por amostragem aleatória simples.

Ao contrário de sua edição de 2019, a PNS 2013 envolveu a coleta de sangue e urina para exames de laboratório.^19^ Um quarto dos conglomerados da PNS 2013 foi selecionado para a coleta de exames laboratoriais, com probabilidade inversamente proporcional à dificuldade de coleta. Como a taxa de não resposta excedeu substancialmente os 20% esperados, os dados foram reponderados para serem representativos em nível macrorregional.^19^

### Variáveis

Neste estudo, calculou-se o risco cardiovascular em dez anos conforme o escore de risco cardiovascular global de Framingham,^9^ PCE (revisão de 2018)^10^ e *Globorisk-LAC*^15^ usando, respectivamente, os pacotes {*CVrisk*} 1.1.1,^20^ {*PooledCohort*} 0.0.2^21^ e {*globorisk*} 1.0.2^22^ para o ambiente de computação estatística R 4.4.0.^23^ Sexo, idade, pressão arterial, colesterol total e colesterol HDL (lipoproteína de alta densidade) foram obtidos das respectivas variáveis. Consideram-se negras as pessoas que relataram pele parda ou preta, e fumantes as pessoas que responderam "sim, diariamente" ou "sim, menos que diariamente".

Como a questão sobre o uso de medicação hipertensiva nas últimas duas semanas só foi feita às pessoas que informaram ter hipertensão, imputou-se a resposta "não" àquelas que descreveram não ter o diagnóstico (fora da gravidez) ou nunca terem tido a pressão arterial medida. Da mesma forma, considerou-se como não tendo diabetes mellitus as pessoas que relataram nunca ter tido sua glicemia medida. Depois desse ajuste, imputou-se o diagnóstico de diabetes mellitus às pessoas cuja hemoglobina glicada na PNS 2013 foi igual ou maior a 6,5%, mesmo se essas não tivessem diagnósticos prévios. Por fim, considerou-se como tendo doença cardiovascular as pessoas que relataram já ter tido diagnóstico de qualquer doença cardíaca ou de acidente vascular cerebral.

### Amostra

O presente estudo incluiu pessoas com idade entre 40 e 74 anos, sem doença cardiovascular conhecida, e sem valores atípicos nem de pressão arterial sistólica, nem de colesterol total (como especificado no desenvolvimento do *Globorisk-LAC*).^15^ Para garantir a validade das comparações, excluímos as pessoas com valores faltantes que impossibilitassem a estimação do risco cardiovascular por qualquer um dos três escores, mesmo quando era possível calcular os demais escores. O peso amostral foi considerado no cálculo de todas as estatísticas, exceto da frequência absoluta.

### Análise estatística

Em sua forma contínua, o risco cardiovascular teve sua distribuição descrita por medianas e intervalos interquartis (IIQ). Para descrever a proporção de participantes nos quais um escore concordava com o outro, considerou-se os escores como concordantes quando o risco mais elevado era menos de 25% superior ao risco menos elevado. Essa forma de comparar as estimativas de risco equivale à diferença entre logaritmos proposta por Bland e Altmann,^24^ só que numa escala mais intuitiva. O ponto de corte de 25% (uma razão de 1,25 entre duas taxas) é uma proposta da metodologia *Grading of Recommendations Assessment, Development and Evaluation* (GRADE) quando não há um consenso sobre qual é o menor tamanho de efeito clinicamente relevante.^25^

O risco cardiovascular também foi estratificado em baixo (menos de 10%), intermediário (10% a menos de 20%) e alto (20% ou mais). Como a estratificação do risco cardiovascular varia entre as diretrizes, optou-se pelo ponto de corte de 20% para risco elevado, com base nas diretrizes da OMS^1^ e da *American Heart Association* (AHA) / *American College of Cardiology* (ACC),^5^ e pelo ponto de corte de 10%, porque corresponde a uma recomendação forte de prescrição de estatina, enquanto a recomendação a partir de 7,5% é condicional.^26^

A distribuição da população brasileira entre os três estratos foi descrita usando frequência relativa (percentuais). Calculou-se a concordância usando tanto o percentual observado de concordância quanto o coeficiente de concordância de primeira ordem de Gwet (AC_1_).^27^ Esse coeficiente tem valores entre −1 e +1, como o coeficiente κ de Cohen, e é mais resistente a paradoxos conhecidos.^27,28^ Em ambos os casos, desconsiderou-se a natureza ordinal da estratificação do risco cardiovascular, de forma que estratos de risco cardiovascular adjacentes foram considerados discordantes, e não parcialmente concordantes.

Como o pacote estatístico {irrCAC}^29^ não considera pesos amostrais ao computar o coeficiente, foi desenvolvido neste estudo código de análise para esse fim. A título de análise de sensibilidade, repetiu-se a estimativa do AC_1_ usando os prontos de corte da AHA/ACC 2018^5^ (5%, 7,5% e 20%), OMS^1^ (10%, 20% e 30%) e *European Society of Cardiology* (ESC) / *European Atherosclerosis Society* (EAS)^3^ (10%, 15% e 30% para eventos fatais + não-fatais, obtidos multiplicando-se por três os pontos de corte de 3%, 5% e 10% para eventos fatais). Todo o código de análise está disponível abertamente.^30^

Os gráficos foram feitos com os pacotes {ggplot2} 3.5.1^31,32^ e {ggalluvial} 0.12.5.^33,34^ Para garantir o entendimento em caso de discromatopsia (ou de impressão em tons de cinza), os gráficos foram feitos usando as escalas de cores de Okabe-Ito,^35^ disponível no R; "dark2" de Brewer,^36^ através do pacote {RColorBrewer} 1.1;^37^ e "cividis",^38^ através do {viridisLite} 0.4.2.^39,40^

### Considerações éticas

A PNS 2013 foi aprovada pelo Conselho Nacional de Ética em Pesquisa (relatório número 328.159). Os participantes assinaram um termo de consentimento antes de a pesquisa ser realizada, e ao final receberam o resultado dos exames laboratoriais realizados.

## Resultados

Dentre os 8.952 participantes do componente laboratorial da PNS 2013, 4.989 (54,0%) tinham entre 40 e 74 anos de idade, 4.781 (51,7%) também tinham dados completos para a estimação do risco cardiovascular e 4.416 (47,0%) não tinham história de doença cardíaca, nem de acidente vascular cerebral. Todos esses 4.416 participantes foram incluídos neste estudo, porque nenhum deles tinha valores atípicos de pressão arterial sistólica ou colesterolemia total.

Os participantes (Tabela 1) eram em sua maioria do sexo feminino (2529; 52,3%), a idade média ± DP foi 53,4 ± 9,4 anos, e praticamente metade (2634; 48,8%) era negra (preta ou parda). Com relação aos fatores clinicamente modificáveis, 796 (17,7%) eram fumantes, 1039 (23,4%) estavam usando anti-hipertensivo e 565 (13,9%) tinham diabetes.

**Tabela 1:** Características demográficas e fatores de risco

Variável	Mediana	Intervalo interquartis
Idade (anos)	52,0	25,0 – 61,0
Colesterol total (mg/dL)	191,0	169,0 – 216,0
Colesterol HDL (mg/dL)	44,0	37,0 –53,0
Pressão arterial sistólica (mmHg)	128,0	116,0 – 140,5

**Figura 1:**
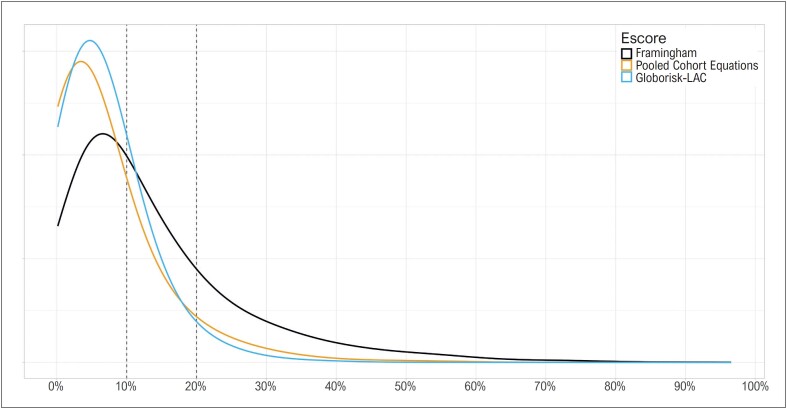
Risco cardiovascular estimado para 10 anos, conforme o escore utilizado para a estimativa.

O risco cardiovascular em dez anos teve mediana de 9,2% (IIQ de 5,1 a 17,8) quando estimado pelo escore de Framingham, 3,6% (IIQ de 1,7 a 8,2) pelo PCE, e 4,7% (IIQ de 2,8 a 8,1) pelo *Globorisk-LAC* (Figura 1). O risco foi considerado baixo em 52,8% dos casos quando estimado pelo escore de Framingham, 79,6% pelo PCE, e 82,2% pelo *Globorisk-LAC*. Adicionalmente, o risco foi considerado intermediário em 20,8% pelo Framingham, 6,5% pelo PCE, e 3,3% pelo *Globorisk-LAC*.

**Figura 2:**
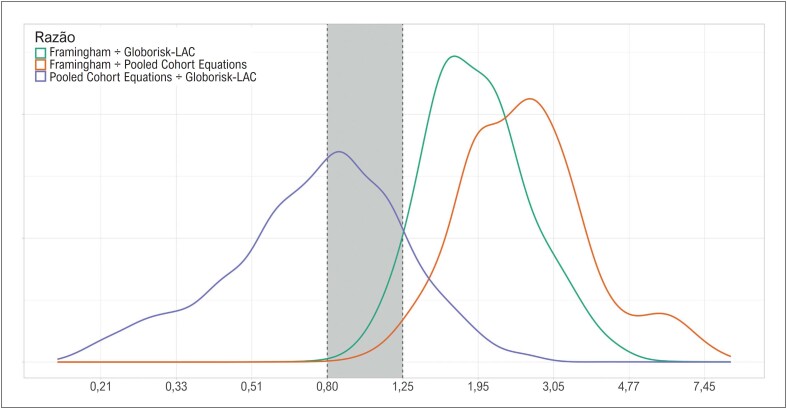
Razão entre o risco cardiovascular estimado para 10 anos entre diferentes escores.

Considerando-se o risco cardiovascular numa escala contínua, o escore de Framingham concordou com o *Globorisk-LAC* na estimação do risco cardiovascular em apenas 6,4% dos casos, e com o PCE em apenas 1,8% (Figura 2). Enquanto isso, o escore PCE concordou com o *Globorisk-LAC* em 34,7% dos casos. O risco estimado pelo Framingham foi substancialmente maior do que aquele estimado pelo *Globorisk-LAC* em 93,5% dos casos, e do que aquele estimado pelo PCE em 98,2% dos casos. Enquanto isso, o risco estimado pelo PCE foi substancialmente menor do que o estimado pelo *Globorisk-LAC* em 50,8% dos casos.

Estratificando-se o risco cardiovascular em baixo, intermediário e alto (Figura Central), o escore de Framingham concordou com o *Globorisk-LAC* em 58,8% dos casos (coeficiente de concordância AC_1_ de 0,454) e com o PCE em 71,6% (AC_1_ de 0,489). Enquanto isso, o escore PCE concordou com o *Globorisk-LAC* em 89,5% dos casos (AC_1_ de 0,874). Observa-se na Figura Central que a maioria dos casos considerados de alto risco pelo Framingham são considerados de risco intermediário pelos outros dois escores. Da mesma forma, a maioria dos casos classificados como de risco intermediário pelo Framingham são considerados de risco baixo pelos outros dois escores.

**Figura Central:**
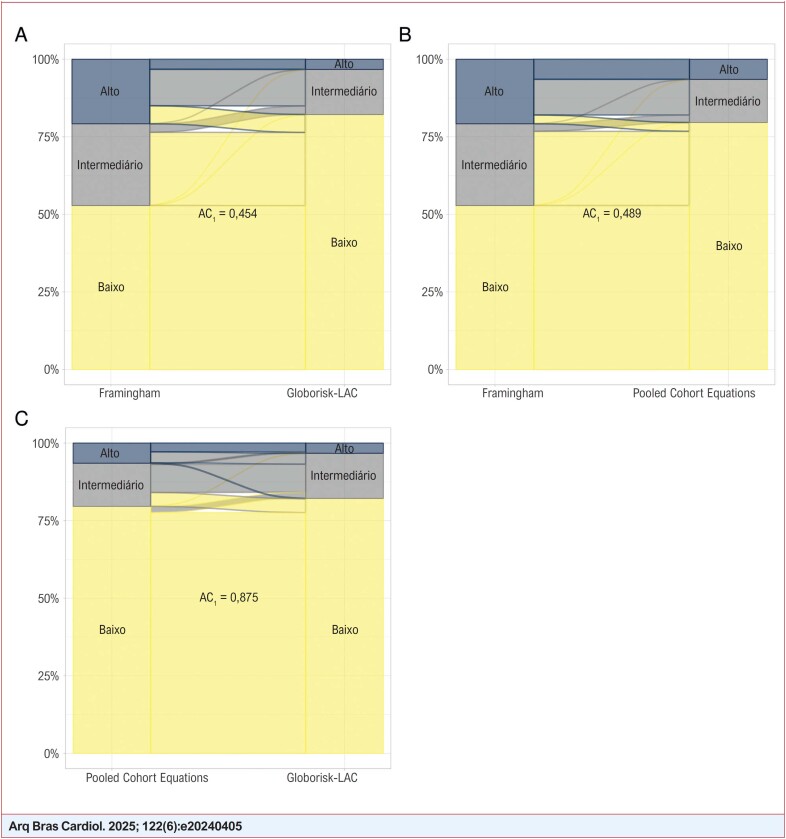
Concordância entre Framingham, Pooled Cohort Equations e Globorisk-LAC da estimação do risco cardiovascular no Brasil, 2013

Diagrama aluvial da concordância entre diferentes escores na estratificação do risco cardiovascular para 10 anos.

A comparação entre os escores PCE e *Globorisk-LAC* na estratificação do risco cardiovascular (Figura Central) difere do que se poderia esperar pela comparação entre os valores dos riscos estimados para dez anos (Figura 2). Mais especificamente, pouco mais da metade dos casos classificados como de alto risco pelo primeiro escore foi de risco intermediário pelo segundo, e um terço dos classificados como de risco intermediário pelo primeiro foi de risco baixo pelo segundo (Figura Central).

Enquanto isso, o risco estimado pelo PCE foi substancialmente menor do que aquele estimado pelo *Globorisk-LAC* em metade dos casos, como demonstrado na Figura 2, sugerindo que, em comparação ao *Globorisk-LAC*, o PCE esteja subestimando o risco nos casos de menor risco, e superestimando nos casos de maior risco. De fato, observa-se na Figura 1 que os valores de risco estimados pelo PCE têm uma distribuição mais ampla do que aqueles estimados pelo *Globorisk-LAC*.

Na análise de sensibilidade, os coeficientes de concordância AC_1_ diminuíram substancialmente com a estratificação do risco da AHA/ACC 2018: 0,191 entre Framingham e *Globorisk-LAC*, 0,228 entre Framingham e PCE e 0.704 entre *PCE* e *Globorisk-LAC*. Por outro lado, os coeficientes praticamente não se alteraram com a estratificação pela OMS (0,476, 0,490 e 0.870, respectivamente) ou pela ESC/EAS 2019 (0,464, 0,486 e 0,848, respectivamente).

## Discussão

Os três escores examinados concordam muito pouco entre si na estimação do risco cardiovascular na população brasileira. Os dois escores mais parecidos entre si foram o PCE e o *Globorisk-LAC*, com um coeficiente de concordância de 0,874. Em comparação ao *Globorisk-LAC*, o PCE subestima o risco cardiovascular das pessoas com menor risco e superestima o risco daquelas com maior risco, causando neste último caso uma mudança na estratificação do risco cardiovascular. Quando comparado a esses dois escores, o de Framingham superestima substancialmente o risco de mais de 95% da população, resultando num coeficiente de concordância de 0,489 e 0,454, respectivamente.

Os mesmos dados foram estudados por Malta et al.,^41^ que encontraram uma prevalência de 44% de risco cardiovascular elevado pelo critério da AHA em 2013;^42^ 38% pelo critério da Sociedade Brasileira de Cardiologia (SBC); e 19% pelo critério do Framingham. O percentual observado de concordância entre o critério da SBC e o da AHA foi de 43% para alto risco e 55% para baixo risco, e entre SBC e Framingham foi 51% para alto risco e 100% para baixo risco.

Em comparação, o presente estudo observou um percentual de 59% de concordância entre Framingham e PCE, considerando simultaneamente três estratos de risco cardiovascular definidos pelos mesmos pontos de corte (< 10%, 10–20%, ≥ 20%) aplicados a todos os escores. Além disso, no presente estudo utilizou-se a versão mais recente, de 2018, do PCE.^10^ Por fim, o foco do presente estudo foi a comparação entre os escores de risco cardiovascular, enquanto Malta et al. focaram na falta de intercambialidade entre os critérios.

Um dos poucos estudos a comparar o Globorisk com outros escores foi o de Osei-Yeboah et al.,^43^ com uma prevalência de risco cardiovascular elevado de 13% pelo Framingham (ponto de corte: 20% em dez anos), 0% pelo Globorisk (ponto de corte: 30%) e 3% pelo PCE2018 (ponto de corte: 20%). O coeficiente κ de Cohen foi 0,008 para a concordância entre Framingham e *Globorisk*, 0,432 entre Framingham e PCE, e 0,020 entre PCE e *Globorisk*. Outro estudo^44^ comparou o *Globorisk* (19% de risco cardiovascular alto ou muito alto) com Framingham (37% de risco alto ou muito alto) em ambulatórios de diabetes mellitus em hospitais de Bangladesh, chegando a um κ de 0,34.

No presente estudo, a concordância entre Framingham e PCE (AC_1_ de 0,489) foi próxima àquela estimada estudo de Osei-Yeboah et al.^43^ enquanto a concordância do *Globorisk-LAC* com Framingham (AC_1_ de 0,454) e PCE (AC_1_ de 0,874) foi muito superior. A diferença se deve, ao menos em parte, ao estudo de Osei-Yeboah et al.^43^ ter adotado um ponto de corte mais elevado (30%) para considerar alto o risco cardiovascular estimado pelo *Globorisk*, resultando em nenhum dos quase 14 mil participantes ter sido considerado de alto risco pelo *Globorisk*. Já o outro estudo^44^ usou os mesmos pontos de corte do presente estudo, resultando em uma estimativa mais próxima à nossa para a concordância entre *Globorisk* e Framingham.

Note-se que ambos os estudos^43,44^ usaram a versão mundial do Globorisk,^13,14^ derivada principalmente de coortes conduzidas em países de alta renda. Até onde sabemos, o presente estudo é o primeiro a estimar a concordância de outros escores com o Globorisk-LAC,^15,17^ que foi derivado de coortes conduzidas na América Latina e Caribe.

Vários estudos compararam o escore de risco de Framingham ao PCE na estratificação do risco cardiovascular. Em estudos realizados em ambulatórios,^45-48^ a prevalência de risco cardiovascular elevado variou de 2% a 50% pelo escore de Framingham e de 5% a 50% pelo PCE, e o coeficiente κ de Cohen variou de 0,049 a 0,745. Já os estudos de base populacional^43,49-51^ encontraram prevalências de risco elevado que variavam de 3% a 19% pelo Framingham e de menos de 1% até 10% pelo PCE, e valores de κ entre 0,29 e 0,55.

Tanto nos estudos com pacientes, quanto naqueles com base populacional, o coeficiente κ de Cohen foi menor nas pesquisas com menores prevalências de risco cardiovascular elevado. Isso ilustra o viés do κ, que subestima a concordância quando uma das categorias tem baixa prevalência.^27,28^ Esse é o motivo pelo qual o presente estudo optou pelo coeficiente AC_1_ de Gwet^27^ como estimando para a concordância na estratificação do risco cardiovascular.

A presente pesquisa foi a primeira a comparar os três escores de risco cardiovascular (Framingham, PCE e *Globorisk-LAC*) usando o método de Bland e Altmann.^24^ Ao menos um outro estudo já usou esse método no contexto do risco cardiovascular, mas para comparar o escore Globorisk com o da OMS.^52^ Diferentemente daquele estudo, optou-se por trabalhar com a razão (em vez da diferença) entre os riscos, e compará-la com um intervalo predefinido (0,8 a 1,25), de forma a poder estimar em que percentual dos casos os escores concordam entre si.

Antes de prosseguir com a interpretação dos achados e a discussão de suas implicações, cumpre ressaltar as limitações deste estudo. O banco de dados laboratoriais da PNS 2013 não contém a variável do conglomerado, impossibilitando que o delineamento da amostragem seja considerado em qualquer forma de inferência estatística, tais como valores p ou intervalos de confiança. O presente estudo usou esses dados mesmo assim, porque a PNS 2013 é o mais recente inquérito nacionalmente representativo que coletou os dados necessários para estimar o risco cardiovascular usando a versão laboratorial dos escores.

Ainda que a baixa taxa de resposta do componente laboratorial tenha o potencial de enviesar as estimativas, espera-se que a reponderação tenha minimizado esse potencial viés. Cumpre lembrar ainda que o presente estudo não verificou a ocorrência dos desfechos preditos, de forma que sua contribuição se limita a comparar os escores com relação à concordância mútua, sem a pretensão de informar qual escore tem a melhor calibração e/ou discriminação.

Por fim, no intuito de manter um número razoável de comparações, este estudo incluiu apenas três escores de risco cardiovascular, deixando de fora, em especial, o escore da OMS e o recém-proposto PREVENT. Como o escore da OMS^12^ faz parte da estratégia HEARTS,^53^ e teve um desempenho superior no estudo de Camargos et al.,^16^ justifica-se aprofundar seu estudo na população brasileira. No caso do PREVENT, com suas inovações conceituais^54^ e desempenho exemplar,^55^ seria necessário recalibrá-lo para a população brasileira antes de cogitar sua adoção. Pode valer a pena, já que nos Estados Unidos, país para o qual foi calibrado, o novo escore discorda do PCE para vários milhões de pessoas.^56^

Cabe agora considerar os motivos pelos quais os diferentes escores de risco cardiovascular discordam tanto entre si quando aplicados à população brasileira. Um motivo é mais óbvio: diferentes escores foram derivados de diferentes coortes e calibrados para diferentes populações. Isso é grande parte do motivo de utilizar-se nos Estados Unidos hoje o PCE, e não o Framingham.^42^ Também é o motivo deste estudo investigar as consequências de uma eventual substituição do Framingham pelo *Globorisk-LAC* nas diretrizes brasileiras de dislipidemia.^4,8^

Outro motivo para a discordância entre os escores são as diferenças na composição dos eventos cardiovasculares preditos. Tanto o PCE quanto o *Globorisk-LAC* focaram nos eventos ateroscleróticos duros: infarto do miocárdio não fatal, morte coronariana e AVE isquêmico ou hemorrágico.^10,15,42^ Já no escore de Framingham, o desfecho inclui ainda insuficiência coronariana, angina, ataque isquêmico transitório, doença arterial periférica e insuficiência cardíaca.^9^

Esses motivos têm consequências para uma eventual atualização das diretrizes clínicas,^4,8^ no que diz respeito à adaptação de recomendações internacionais. Por exemplo, hoje se recomenda nos Estados Unidos a profilaxia medicamentosa da doença aterosclerótica a partir de 7,5%^5^ ou 10%^26^ de risco cardiovascular em dez anos, estimado através do PCE. No que diz respeito à interpretação dos escores de risco cardiovascular, os mesmos pontos de corte poderiam ser usados no Brasil se o risco for estimado pelo *Globorisk-LAC*. Não se pode dizer o mesmo sobre o escore de Framingham, cuja publicação^9^ não traz uma versão específica para doença aterosclerótica. Ademais, cumpre lembrar que o escore de Framingham nunca foi recalibrado para a população brasileira.^11^

## Conclusão

Na população brasileira, o risco cardiovascular estimado para dez anos difere substancialmente entre os escores de risco de Framingham, do PCE e do *Globorisk-LAC*. A adequação à realidade brasileira e a similaridade de desfechos com PCE fazem do *Globorisk-LAC* uma ótima opção para suceder o Framingham na próxima atualização das diretrizes brasileiras de dislipidemia e prevenção de aterosclerose, bem como dos respectivos protocolos clínicos e diretrizes terapêuticas.

## Data Availability

Os conteúdos estão disponíveis no DOI https://doi.org/10.5281/zenodo.13948103.
