# Supplementary material for: Concordância entre Framingham, Pooled Cohort Equations e Globorisk-LAC da estimação do risco cardiovascular no Brasil, 2013
Source: Arq Bras Cardiol. 2025 Jul 1;122(6):e20240405. [Article in Portuguese] doi: 10.36660/abc.20240405 (PMC12269898; doi:10.36660/abc.20240405)
Supplement: Translation

# Translation: Agreement between Framingham, Pooled Cohort Equations, and Globorisk-LAC in the Estimation of Cardiovascular Risk in Brazil, 2013

**DOI:** 10.36660/abc.20240405i

**Authors:** Leonardo Ferreira Fontenelle, Thiago Dias Sarti, Gabriela Callo Quinte, Ana Paula Santana Coelho Almeida, José Geraldo Mill

**Affiliations:** 1 Vitoria ES Brazil Universidade Federal do Espírito Santo (UFES) Departamento de Medicina Social, Vitoria, ES – Brazil; 2 Vitoria ES Brazil Universidade Federal do Espírito Santo (UFES) Programa de Pós-Graduação em Saúde Coletiva, Vitoria, ES – Brazil; 3 Vitoria ES Brazil Universidade Federal do Espírito Santo (UFES) Programa de Pós-Graduação em Ciências Fisiológicas, Vitoria, ES – Brazil

**Keywords:** Heart Disease Risk Factors, Brazil, Risk Assessment

## Abstract

**Background:**

The Framingham risk score and Pooled Cohort Equations (PCE) have never been recalibrated for the Brazilian population. In contrast, the Globorisk-LAC score was recently derived using a methodology analogous to the PCE and has been recalibrated for Latin American countries.

**Objectives:**

To describe the agreement between the Framingham, PCE, and Globorisk-LAC scores in estimating the 10-year cardiovascular risk in the Brazilian population.

**Methods:**

This cross-sectional study used the three scores to estimate cardiovascular risk in participants aged 40 to 74 years without a history of cardiovascular disease based on data from the 2013 National Health Survey (PNS). The agreement was estimated as (i) the percentage of participants in which the risk estimated by one score was between 0.80 and 1.25 times the risk estimated by another score and (ii) based on the Gwet's agreement coefficient (AC_1_) according to risk categories (low, intermediate, and high).

**Results:**

A total of 4,416 participants were included from 8,952 participants from the PNS with a laboratory component. The median (interquartile range) of the estimated 10-year cardiovascular risk was 9.2% (5.1 to 17.8) according to the Framingham, 3.6% (1.7 to 8.2) according to the PCE, and 4.7% (2.8 to 8.1) according to the Globorisk-LAC. The risk estimated using the Framingham agreed with the Globorisk-LAC and PCE in 6.4% and 1.8% of the cases, respectively, whereas the PCE and Globorisk-LAC agreed in 34.7% of the cases. When considering the risk stratification, the respective AC_1_ values were 0.454, 0.489, and 0.874.

**Conclusions:**

The three cardiovascular risk scores showed low levels of agreement with each other. The reasons for this disagreement suggests that Globorisk-LAC is a strong candidate to replace the Framingham in the Brazilian guidelines for dyslipidemia.

## Introduction

Cardiovascular guidelines^1-5^ widely recommend cardiovascular risk stratification as a tool to guide the pharmacological management of dyslipidemia and other risk factors. Typically, this stratification initiates by assigning participants with established cardiovascular disease to higher-risk categories. For those without established disease, the stratification involves applying clinically relevant cutoff points to the estimated 10-year risk of cardiovascular event obtained from a prognostic risk score.

As with other prognostic models, the calibration and discrimination of cardiovascular risk scores should be validated in the population to which they will be applied. For example, the Prospective Urban Rural Epidemiology study found low-and middle-income countries to have a higher frequency of cardiovascular events, despite having lower risk scores than in high-income countries.^6^ Because of this, scores are often recalibrated for the populations to which they will be applied.^6,7^ The ease of recalibrating a given score is important for its recommendation by clinical guidelines.^1,3^

The recent Brazilian guidelines for dyslipidemia^4,8^ recommend the Framingham global cardiovascular risk score.^9^ In contrast, American guidelines recommend the Pooled Cohort Equations (PCE),^5^ which combine the Framingham with other cohorts from the United States, recalibrating the model to reflect the contemporary population.^10^ However, the Framingham and PCE have not been recalibrated for the Brazilian population.^11^ The only cardiovascular risk scores calibrated for the Brazilian population are the World Health Organization (WHO),^12^ Globorisk,^13,14^ and Globorisk-LAC.^15^ In a Brazilian study without representativity of the general population,^16^ all scores evaluated (including the WHO and Globorisk-LAC) overestimated the cardiovascular risk despite having a discrimination ability similar to that observed in the original validations; among them, the WHO score had a smaller degree of overestimation. In contrast, the Globorisk-LAC has the advantage of being derived from Latin American cohorts,^17^ and its calibrated version performed similarly or slightly better than the WHO model when applied to the same population-based cohorts.^15^

As literature lacks studies examining the agreement between the Globorisk-LAC and other cardiovascular risk scores, it remains unclear the impacts of using the Globorisk-LAC instead of the Framingham in the Brazilian guidelines for dyslipidemia.^4,8^ This study aimed to evaluate the agreement between the Framingham, PCE, and Globorisk-LAC in estimating the 10-year cardiovascular risk in the Brazilian population.

## Methods

### Study design

This descriptive cross-sectional study used data from the 2013 National Health Survey (PNS),^18^ which was a three-stage household survey conducted between August and December of 2013. Census tracts (clusters) were randomly selected using a probability proportional to the number of private permanent households. Households were selected within census tracts, and one resident aged _≥_ 18 years within each household, via simple random sampling.

Unlike the 2019 edition, the 2013 PNS included blood and urine for laboratory testing.^19^ One-quarter of the clusters were chosen for laboratory testing, with a probability inversely proportional to the difficulty of collecting the specimens. As the non-response rate was substantially higher than the expected 20%, data were reweighted to ensure macro-regional representativeness.^19^

### Variables

The 10-year cardiovascular risk was estimated according to the the Framingham global cardiovascular risk score,^9^ the 2018 PCE revised,^10^ and the Globorisk-LAC,^15^ using the {CVrisk} 1.1.1,^20^ {PooledCohort} 0.0.2,^21^ and {globorisk} 1.0.2^22^ packages for the R statistical computing environment, version 4.4.0.^23^ Gender, age, blood pressure, total cholesterol, and high-density lipoprotein cholesterol were derived from the respective survey variables. Participants who self-identified as brown or black were categorized as black. Smokers included participants who answered "yes, daily" or "yes, less than daily". Because the question regarding recent antihypertensive medication use was only asked to the participants who reported a diagnosis of hypertension, a "no" response was imputed for those denying a diagnosis (excluding during pregnancy) or reporting never having their blood pressure measured. Similarly, participants who had never undergone blood glucose testing were classified as not having diabetes mellitus. Subsequently, a diagnosis of diabetes mellitus was imputed for participants with glycated hemoglobin levels ≥ 6.5% in the 2013 PNS, regardless of self-reported diagnosis. Participants who reported a prior diagnosis of any heart disease or stroke were considered to have cardiovascular disease.

### Sample

The study included participants aged 40 to 74 without known cardiovascular disease and with systolic blood pressure and total cholesterol values within the range specified by the Globorisk-LAC model.^15^ To ensure the validity of comparisons, participants with missing values that precluded the risk calculation for any of the three scores were excluded. Sample weights were applied to all statistical analyses, except for absolute frequency.

### Statistical analysis

Cardiovascular risk was summarized using medians and interquartile ranges (IQR). The agreement between scores was evaluated by calculating the proportion of participants whose higher estimated risk was less than 25% greater than the lower. This approach is analogous to comparing logarithmic differences, as proposed by Bland & Altmann,^24^ but presented on a more intuitive scale. The 25% cutoff point (i.e., ratio of 1.25 between two rates) is proposed by the GRADE methodology when there is no consensus regarding the lower clinically relevant effect size.^25^

The cardiovascular risk was stratified into low (< 10%), intermediate (10% to < 20%), and high (≥ 20%). As the cardiovascular risk stratification varies between guidelines, the 20% threshold for high-risk followed the WHO^1^ and American Heart Association (AHA)/American College of Cardiology (ACC) guidelines,^5^ while the 10% threshold followed a strong recommendation for statin prescription, since the 7,5% threshold is a conditional recommendation.^26^ The distribution of the Brazilian population among the three strata was described using relative frequency (percentage). The agreement was calculated using the observed percentage agreement and Gwet's first-order agreement coefficient (AC_1_);^27^ the latter ranges from −1 to +1 (similar to Cohen's κ coefficient) and is more robust to known paradoxes.^27,28^ In both cases, the ordinal nature of cardiovascular risk stratification was not considered, and adjacent categories were treated as discordant and not partially concordant. As the {irrCAC} package^29^ does not support sampling weights, custom R code to compute AC_1_ using sample weights was developed. For the sensitivity analysis, the estimated AC_1_ was recalculated using cutoff points from the 2018 AHA/ACC^5^ (5%, 7.5%, and 20%), WHO^1^ (10%, 20%, and 30%), and European Society of Cardiology (ESC)/European Atherosclerosis Society (EAS)^3^ (10%, 15%, and 30% for fatal + nonfatal events obtained by multiplying by three the cutoff points of 3%, 5%, and 10% for fatal events). The code used for analysis is openly available.^30^

Figures were generated using the {ggplot2} 3.5.1^31,32^ and {ggalluvial} 0.12.5^33,34^ packages and the following color palettes designed for accessibility in cases of dyschromatopsia or grayscale printing: Okabe-Ito^35^ (available from R), "dark2" from Brewer^36^ ({RColorBrewer} 1.1 package),^37^ and "cividis"^38^ ({viridisLite} 0.4.2).^39,40^

### Ethical considerations

The 2013 PNS was approved by the National Research Ethics Committee (number 328,159). The participants signed an informed consent form before participating and received their laboratory test results after the study.

## Results

Among the 8,952 participants in the laboratory component of the 2013 PNS, 4,989 (54.0%) were aged between 40 and 74 years, 4,781 (51.7%) had complete data for cardiovascular risk estimation, and 4,416 (47.0%) had no history of heart disease or stroke. All 4,416 participants were included in this study, as none presented atypical values for systolic blood pressure or total cholesterol.

The participants (Table 1) were predominantly female (n = 2,529; 52.3%), with a mean ± SD age of 53.4 ± 9.4 years. Nearly half (n = 2,634; 48.8%) self-identified as black or brown. Regarding modifiable clinical risk factors, 796 (17.7%) were smokers, 1,039 (23.4%) were using antihypertensive medications, and 565 (13.9%) had diabetes.

**Table 1:** Demographic characteristics and cardiovascular risk factors of the study population

Variable	Median	Interquartile range
Age (years)	52.0	25.0 to 61.0
Total cholesterol (mg/dL)	191.0	169.0 to 216.0
HDL cholesterol (mg/dL)	44.0	37.0 to 53.0
Systolic blood pressure (mmHg)	128.0	116.0 to 140.5

The median 10-year cardiovascular risk was 9.2% (IQR 5.1% to 17.8%) when estimated using the Framingham, 3.6% (IQR 1.7% to 8.2%) using the PCE, and 4.7% (IQR 2.8% to 8.1%) using the Globorisk-LAC (Figure 1). The risk was considered low in 52.8% of participants on the Framingham, 79.6% on the PCE, and 82.2% on the Globorisk-LAC. An intermediate risk was found in 20.8% of the individuals using the Framingham, 6.5% using the PCE, and 3.3% using Globorisk-LAC.

**Figure 1:** Estimated 10-year cardiovascular risk across different risk scores.

Considering cardiovascular risk as a continuous variable, the Framingham score agreed with the Globorisk-LAC and PCE in 6.4% and 1.8% of participants, respectively (Figure 2). In contrast, the PCE score agreed with the Globorisk-LAC in 34.7% of the cases. The Framingham produced substantially higher risk estimates than the Globorisk-LAC with each other in 93.5% of participants, and higher than the PCE in 98.2%. Meanwhile, the PCE produced substantially lower risk estimates than the Globorisk-LAC in 50.8% of the participants.

**Figure 2:** Ratio of estimated 10-year cardiovascular risk between different scores.

When stratifying cardiovascular risk into low, intermediate, and high (Central Illustration), the Framingham agreed with the Globorisk-LAC in 58.8% of the participants (AC_1_ = 0.454) and with the PCE in 71.6% (AC_1_ = 0.489). The highest agreement was observed between PCE and Globorisk-LAC (89.5%; AC_1_ = 0.874). As shown in the Central Illustration, most participants classified as high risk by the Framingham were classified as intermediate risk by the other two scores. Similarly, most participants classified as intermediate risk by the Framingham were categorized as low risk by the other two scores.

**Central Illustration:** Agreement between Framingham, Pooled Cohort Equations, and Globorisk-LAC in the Estimation of Cardiovascular Risk in Brazil, 2013

Alluvial diagram illustrating the agreement between different scores in the 10-year cardiovascular risk stratification.

The comparison between PCE and Globorisk-LAC risk stratification (Central Illustration) deviated from what could be expected by comparing the values of 10-year risk estimates (Figure 2). Specifically, just over half of the participants classified as high risk by the PCE were classified as intermediate risk by Globorisk-LAC, and one-third in the PCE intermediate risk were classified as low risk by the Globorisk-LAC (Central Illustration). However, the PCE produced lower risk estimates than Globorisk-LAC in half of the cases (Figure 2), suggesting that PCE may underestimate the risk in lower-risk participants while overestimating it in those who are higher-risk. Indeed, Figure 1 shows a wider distribution of risk estimates in the PCE compared with the Globorisk-LAC.

In the sensitivity analysis, the AC_1_ decreased when using the 2018 AHA/ACC risk stratification thresholds: 0.191 between the Framingham and Globorisk-LAC, 0.228 between the Framingham and PCE, and 0.704 between PCE and Globorisk-LAC. In contrast, AC_1_ values remained relatively stable when using the WHO (0.476, 0.490, and 0.870, respectively) or 2019 ESC/EAS risk stratification (0.464, 0.486, and 0.848, respectively).

## Discussion

The three cardiovascular risk scores analyzed in this study showed a low agreement with each other for estimating cardiovascular risk in the Brazilian population. The highest agreement was observed between the PCE and Globorisk-LAC (AC_1_ of 0.874). Compared with the Globorisk-LAC, the PCE underestimated the cardiovascular risk in participants with lower risk and overestimated in those with higher risk; the latter caused a change in cardiovascular risk stratification. Moreover, the Framingham overestimated the cardiovascular risk in over 95% of participants compared with the PCE (AC_1_ of 0.489) and Globorisk-LAC (AC_1_ of 0.454).

Malta et al.^41^ analyzed the same dataset and reported a 44% prevalence of high cardiovascular risk based on the 2013 AHA,^42^ 38% using the criteria of the Brazilian Society of Cardiology (SBC), and 19% using the Framingham. In the study, the observed agreement between the SBC and AHA criteria was 43% for high risk and 55% for low risk, whereas an agreement of 51% for high risk and 100% for low risk was found between the SBC and Framingham.

The present study observed a 59% agreement between the Framingham and PCE by simultaneously considering the three risk categories defined by the same cutoff point (i.e., < 10%, 10 to 20%, ≥ 20%), uniformly applied across the scores. Additionally, the analysis used the most recent version of the 2018 PCE.^10^ The main objective was to compare the risk scores, whereas Malta et al. focused on the limited interchangeability of the classification criteria.

One of the few studies comparing the Globorisk with other scores was conducted by Osei-Yeboah et al.,^43^ who reported a 13% prevalence of high cardiovascular risk using the Framingham (cutoff point of 20% in 10 years), 0% using the Globorisk (cutoff point of 30%), and 3% using the 2018 PCE (cutoff point of 20%). Cohen's κ coefficient was 0.008 for the agreement between Framingham and Globorisk, 0.432 between the Framingham and PCE, and 0.020 between PCE and Globorisk scores. Another study^44^ compared the Globorisk (19% high or very high cardiovascular risk) with Framingham (37% high or very high cardiovascular risk) in outpatient clinics for diabetes mellitus in Bangladesh hospitals and observed a Cohen's κ of 0.34.

In our study, the agreement between Framingham and PCE (AC_1_ = 0.489) was similar to that reported by Osei-Yeboah et al.,^43^ whereas the agreements between the Globorisk-LAC and Framingham (AC_1_ = 0.454) and PCE (AC_1_ = 0.874) were considerably higher. This discrepancy is partly explained by the higher cutoff point (30%) used by Osei-Yeboah et al.^43^ to define high cardiovascular risk according to the Globorisk, which led to none of the nearly 14,000 participants being classified as high risk. In contrast, the other study^44^ used the same cutoff point and found an agreement between Globorisk and Framingham similar to that observed in our study.

Notably, both studies^43,44^ used the global version of the Globorisk^13,14^ derived primarily from cohorts conducted in high-income countries. To our knowledge, the present study was the first to estimate agreements between other risk scores and the Globorisk-LAC,^15,17^ which was specifically developed using cohorts from Latin America and the Caribbean.

Several studies have compared the Framingham with the PCE for cardiovascular risk stratification. In studies conducted in outpatient clinics,^45-48^ the prevalence of high cardiovascular risk ranged from 2% to 50% using the Framingham and from 5% to 50% using PCE, with Cohen's κ values ranging from 0.049 to 0.745. In contrast, population-based studies^43,49-51^ reported a high-risk prevalence ranging from 3% to 19% using the Framingham and less than 1% to 10% using the PCE (Cohen's κ between 0.29 and 0.55).

A low Cohen's κ was observed in clinical and population-based studies with a lower prevalence of high cardiovascular risk. This illustrates the Cohen's κ bias, which underestimates the agreement when one of the categories has a low prevalence.^27,28^ This is why our study opted for Gwet's AC_1_^27^ as the estimand for the agreement in cardiovascular risk stratification.

This study was the first to compare the Framingham, PCE, and Globorisk-LAC scores using the Bland & Altmann method.^24^ At least one previous study used this method to compare the Globorisk and WHO scores.^52^ Nevertheless, our study used the ratio rather than the difference between risk estimates and compared it with a predefined range (0.8 to 1.25) to estimate the percentage of cases in which the scores agreed with each other.

Some limitations must be acknowledged. The 2013 PNS laboratory testing database lacked information on clusters, precluding statistical inferences involving confidence intervals or p-values. Nevertheless, this database is the most recent population-representative survey in Brazil that included the necessary laboratory data to estimate cardiovascular risk. Although the low response rate of the laboratory component may have introduced bias, reweighting data likely mitigated this effect. It should also be emphasized that the present study neither verified the predicted outcomes nor determined which scores had superior calibration and discrimination. Instead, the study was limited to evaluating the agreement between cardiovascular risk scores.

To avoid excessive comparisons, the analysis was limited to three cardiovascular risk scores, excluding the WHO score and the recent PREVENT tool. As the WHO score^12^ is part of the HEARTS strategy^53^ and demonstrated superior performance in the study by Camargos et al.,^16^ more studies are needed with the Brazilian population. Recalibration of the PREVENT tool, which incorporated innovative concepts^54^ and showed exemplary performance,^55^ would also be required before it could be considered for use in Brazil. This effort may be worthwhile, as the new tool disagreed with the PCE in estimating the cardiovascular risk of millions of people in the United States.^56^

The substantial disagreement among cardiovascular risk scores when applied to the Brazilian population may be justified by two main factors. First, each score was derived from different cohorts and calibrated for distinct populations. This explains, for example, why the PCE is now used in the United States instead of the Framingham^42^ and why our study evaluated the consequences of replacing the Framingham with Globorisk-LAC in Brazilian guidelines for dyslipidemia.^4,8^

Second, the scores differ in the composition of predicted cardiovascular events. The PCE and the Globorisk-LAC estimate the risk of hard atherosclerotic events: nonfatal myocardial infarction, coronary death, and ischemic or hemorrhagic stroke.^10,15,42^ In contrast, the Framingham also includes coronary insufficiency, angina, transient ischemic attack, peripheral arterial disease, and heart failure.^9^

These two factors have important consequences for a potential update of clinical practice guidelines,^4,8^ particularly regarding adapting international recommendations to the Brazilian context. For example, based on the PCE, the pharmacological prevention of atherosclerotic disease in the United States is currently recommended at thresholds of 7.5%^5^ or 10%^26^ of 10-year cardiovascular risk. Regarding the interpretation of cardiovascular risk scores, the same cutoff points could be used in Brazil if the risk is estimated using the Globorisk-LAC. On the other hand, this could not be applied to the Framingham, which does not have a version specifically designed for atherosclerotic risk^9^ and has never been recalibrated for the Brazilian population.^11^

## Conclusion

In the Brazilian population, the estimated 10-year cardiovascular risk substantially differed among the Framingham, PCE, and Globorisk-LAC scores. Given its derivation from Latin American cohorts and its similarity to PCE estimates, the Globorisk-LAC is an excellent option to succeed the Framingham in future updates of the Brazilian Guidelines for Dyslipidemia and Atherosclerosis Prevention, including the associated Clinical Protocols and Therapeutic Guidelines.

## Data Availability

The content is available at the DOI https://doi.org/10.5281/zenodo.13948103.
